# Healthcare student-patient relationship and the quality of the clinical learning environment – a cross-sectional study

**DOI:** 10.1186/s12909-021-02676-x

**Published:** 2021-04-22

**Authors:** Arja Suikkala, Leena Timonen, Helena Leino-Kilpi, Jouko Katajisto, Camilla Strandell-Laine

**Affiliations:** 1 Diaconia University of Applied Sciences, Kyläsaarenkuja 2, FI- 00580 Helsinki, Finland; 2 Department of Nursing Science, University of Turku, Turku, Finland; 3 Helsinki University Hospital, Helsinki, Finland; 4 Turku University Hospital, Turku, Finland; 5 Department of Mathematics and Statistics, University of Turku, Turku, Finland; 6 Novia University of Applied Sciences, Turku, Finland

**Keywords:** Clinical learning environment, Cross-sectional study, Healthcare students, Student-patient relations, Supervision, Teacher

## Abstract

**Background:**

Relationships with patients are seen as the core component of establishing the quality of patient-centred care and promoting patients’ autonomy and relevant use of services. A clinical learning environment that emphasizes relationship-based healthcare is essential for encouraging future healthcare professionals to work in partnership with patients. There is also broad agreement that the insight of patients should be used actively in healthcare students’ clinical learning. The aim of this study was to describe healthcare students’ perceptions of their relationship with patients and the quality of the clinical learning environment and to identify factors associated with both of these.

**Methods:**

A cross-sectional survey using an electronic questionnaire was applied to collect data from 1644 Finnish healthcare students, mostly nursing students, between January 2018 and May 2018. The data were analysed statistically using descriptive statistics, Spearman’s correlation coefficients, and multifactor analysis of variance.

**Results:**

Students perceived the level of the student-patient relationship and the role of the teacher as good while pedagogical atmosphere, premises of care, premises of learning, and supervisory relationship were perceived to be at very good level. The correlations between the student-patient relationship and all clinical learning environment dimensions were perceived as moderate. Furthermore, a number of student-related factors associated with the student-patient relationship and the quality of the clinical learning environment were detected.

**Conclusions:**

In this study, the rarely explored perspective of the student-patient relationship within the context of the clinical learning environment was included. The student-patient relationship and the quality of the clinical learning environment were perceived as good by the students, with a number of determining factors affecting these perceptions. Giving the student-patient relationship a role in clinical education may be conducive to students’ learning with the patient in focus, and may thus promote the competence needed in the rapidly evolving healthcare environment and the changing scope of clinical practice.

## Background

Health professional education aims to produce sufficient, adequately distributed and qualified health professionals responsive to the health needs of populations. At the same time, the global shortage and maldistribution of healthcare professionals is prompting both educational institutions and healthcare organisations to create enabling environments for caring and learning in order to enhance the motivation, attraction and retention of a well-trained future healthcare workforce that is able to adapt to the diverse and changing needs and expectations of individual patients [1–3].

The healthcare student-patient relationship constitutes the core of competence-based health care education [4, 5]. The idea of learning with rather than about patients is emerging and spreading, calling for patients to adopt more active roles and bring their expertise and voice into healthcare education. Focusing learning on the student-patient relationship offers patients a variety of opportunities for educational and evaluation-related roles that can enrich students’ clinical learning and evaluation and thus promote students to provide holistic comprehensive care. Thus, all patients with experience and capacity can participate in learning relationships with students and thereby improve the overall quality of the education. However, within the context of student-patient relationships it is paramount to ensure patient safety and patients’ informed consent of whether or not to participate in students’ learning and assessment [5, 6].

In the literature, different student-patient relationships have been described where patients’ level of participation has ranged from a passive role in the learning activities initiated by supervisors to more active and collaborate roles facilitating students’ learning [6–8]. Facilitative student-patient relationships engage students and patients in mutually beneficial dialogue and shared learning that benefits both. This means that the students listen and respond to the patients’ concerns and wishes. Through knowing the patients personally students encourage and act as advocates for the patients. The patients, in turn, have expertise of their own situation and participate in the learning relationship with the students by expressing opinions and providing the students with information and advice on matters related to their experienced state of health and care. By providing valuable feedback on students’ performance from the patient’s point of view patients have an opportunity to bolster students’ confidence and competency development [8, 9].

Patient relationships are determined by the clinical learning environment (CLE) in terms of the type, quality and amount of communication, attitudes and behaviours displayed by students, patients, supervisors, and the interprofessional team in the practicum placement. Students succeed in creating positive encounters with patients, and a good atmosphere, with a supervisor in a supportive role, thus promotes patients’ active role in contributing to student learning [6, 7]. The student supervision is provided by clinical staff supervisors who balance between the safe care of patients and the learning of a student or group of students assigned to a personal supervisor or supervisors who vary according to shift or place of work [10–14]. As the clinical environment can be stressful for students as a result of both new relationships and a new environment, feeling welcomed and accepted as an equal member in interprofessional teamwork is of great importance [12, 15, 16].

Individualised supportive supervision and continuous feedback based on students’ learning needs, goals, stage of learning and the contribution of a teacher often hired by educational institutions have been found to be crucial for positive learning experiences [10, 17, 18]. Although teachers’ pedagogical role in the integration of theory and practice and their support and guidance for students’ professional development have been well acknowledged, their actual presence in the clinical placements has been gradually replaced by increasing use of information and communication technology for remote student-teacher interaction [19, 20].

Meaningful patient relationships with positive CLE experiences can determine students’ achievement of learning outcomes, self-confidence, and even their intention to remain in the healthcare profession [8, 21]. Students who are actively involved in establishing positive interpersonal relationships and adopt responsibility for their own learning facilitated by an individual relationship with a supervisor and a teacher, are often more satisfied with the CLE than students who are given more of an observational role [12, 13, 15, 21, 22]. Furthermore, longer practicum periods and the time spent on direct patient contacts are seen as crucial for providing positive learning experiences that foster students’ professional confidence and competence in planning, delivering and evaluating care in partnership with patients [13, 23, 24].

Increasingly, there are educational initiatives where patients are involved in the clinical training of healthcare professionals to inspire the supervision of students, with the patient in focus. Nevertheless, there is scarcity of research on the student-patient relationship in the context of CLE [6, 13, 25].

The aim of this study was to describe healthcare students’ perceptions of their relationship with patients and the quality of CLE and to identify factors associated with both of these.

The following research questions were addressed:

1. What are healthcare students’ perceptions of the student-patient relationship?
2. What are healthcare students’ perceptions of the quality of the CLE?
3. What is the association between the patient relationship and the quality of the CLE?
4. What factors are associated with the student-patient relationship and the quality of the CLE?

## Methods

### Design, sample and data collection

This study used cross-sectional survey data collected from healthcare students between 1st January and 31st May 2018 in one university hospital district. The university hospital district was made up of five hospital areas and 23 hospitals representing the largest hospital district in Finland. The data collected using an electronic questionnaire by the hospital district was part of the quality assessment and development of clinical learning environments and supervision. All healthcare students who participated in a clinical placement in the Spring 2018 semester were asked to complete the questionnaire during the last week of their clinical practicum. Instructions for answering the survey were given by the students’ supervisors.

The data consisted of a total of 1664 responses from healthcare students who volunteered to answer the questionnaire. The data represented 33.0% of all healthcare students (*N* = 5042) in the university hospital district who were practising in clinical placements in the study year 2018. The students, most of whom were studying towards registered nurse qualification, were completing a university of applied sciences degree programme defined in the Bologna Declaration and in the EU directives [26–28]. A minority of the students were studying in a social and healthcare degree programme provided by vocational institutions [29–31]. In most cases, the students had reserved the clinical placements, which had been confirmed by their teachers as appropriate for the students’ stage of training. The students were thus completing their clinical practicum in a variety of specialty fields.

### Instrument

The data were collected using the Student Feedback Questionnaire. The Student Feedback Questionnaire was developed for the purposes of national benchmarking in Finland [32] to collect benchmark data of the student-patient relationship, the quality of the CLE, and information about students’ background (13 items).

The Student-Patient Relationship (SPR) Scale included 13 items measuring a facilitative student-patient relationship and thus offering a more comprehensive understanding of the CLE where the patients are in focus. The items were based on a literature review [9], interview study [8] as well as testing with both nursing students and patients [33–35].

For the Student Feedback Questionnaire, the CLE was measured by using the CLES+T Scale that had been modified suitable for healthcare students together with the developer of the CLES+T Scale. For that purpose, two items of the original CLES+T Scale describing the ward manager as a team member and the ward as a good learning environment were deleted. In contrast, three items describing well-organised basic familiarization, use of patient cases in the student supervisory process and supervisor’s supervision skills that support learning were added [36]. In this study, The CLES+T Scale included the following dimensions: pedagogical atmosphere (7 items), premises of care (4 items), premises of learning (7 items), supervisory relationship (8 items), and role of the teacher (9 items) [32, 37, 38]. The original CLES+T Scale has been used in several different languages, cultures and contexts mostly for nursing students [11, 18, 22, 24, 39, 40], but more recently, for other health professional students as well [36, 41–43].

In both scales, an 11-point Likert scale (0 = strongly disagree, 10 = strongly agree) was used. In the interpretation of descriptive statistics, the mean values were divided into four equal parts in order to describe the level of students’ perceptions: very weak (0–2.0), weak (2.1–4.0), satisfactory (4.1–6.0), good (6.1–8.0), and very good (to be pursued 8.1–10).

### Data analysis

The data were analysed using SPSS 22.0 (SPSS Inc., Chicago, USA) software and described by using frequencies, percentages, means and standard deviations (SD). Dependencies between the student-patient relationship (SPR Scale) and CLE (CLES + T Scale) were examined with Spearman’s rho correlation coefficient. This was the first study examining the student-patient relationship and the quality of the CLE in the same study. Therefore, multifactor analysis of variance was used to find a more comprehensive understanding of the background factors associated with the student-patient relationship (SPR Scale) and the quality of CLE in terms of all five dimensions of the CLES + T Scale.

### Ethical considerations

The study followed the principles of research ethics [44, 45] and the General Data Protection Regulation [46] (Regulation EU 2016/679). Research permission was obtained from the university hospital district according to the organization’s ethical committee policies. The informed consent page of the questionnaire contained the legally required data protection information to allow students to make an informed decision of whether to participate in the survey or not. By answering the questionnaire and pressing the submit button, students gave informed consent for the use of their answers for both benchmarking and research purposes. Students completed the electronic questionnaire completely anonymously via a secure connection. The data were processed as confidential and the privacy of the participants was protected.

## Results

### Student characteristics

Of the 1644 students, most were at least 25 years old, studying in a degree programme leading to registered nurse qualification, and in their second and third year of studies. The students were mostly undertaking a clinical placement of four to more than 6 weeks. In most cases, the students had a mid-term feedback session. Most of the students achieved their clinical learning goals and received supervision that supported their professional development at least fairly well. However, only about two thirds perceived that their theoretical studies supported learning in clinical practice very well or fairly well. A detailed description of the students’ characteristics is presented in Table 1.

**Table 1:** Characteristics of the healthcare students (*n* = 1656 − 1664)

Socio-demographic and background factors related to the clinical practicum	Students	
	n	%
Age in years		
< 20	25	1.5
20–24	688	41.3
25–29	474	28.5
≥ 30	477	28.7
Previous professional qualifications^a^		
Yes	1061	63.9
No	600	36.1
Current phase of studies		
First year	211	12.7
Second year	584	35.1
Third year	646	38.8
Fourth year	223	13.4
Degree programme		
Registered nurse	881	52.9
Registered nurse, public health nurse	182	10.9
Registered nurse, midwife	158	9.5
Radiographer	122	7.3
Registered nurse, paramedics	103	6.2
Biomedical laboratory scientist	60	3.6
Practical nurse for social and healthcare^b^	49	2.9
Physiotherapist	48	2.9
Occupational therapist	17	1.0
Something else (e.g. audiometrist, podiatrist, dental hygienist)	44	2.7
Duration of clinical practicum		
≤ 3 weeks or less	260	15.6
4–5 weeks	749	45.0
≥ 6 weeks	655	39.4
Occurrence of supervision		
A named personal supervisor and a working relationship	1249	75.1
A named personal supervisor, but not a working relationship	147	8.8
The supervisor changed during the placement	13	0.8
No named supervisor at all	28	1.7
Supervisor varied according to shift or place of work	178	10.7
A group supervisor rather than an individual supervisor	49	2.9
Discussion about learning goals with a named supervisor^a^		
Yes	1592	96.1
No	64	3.9
Having a mid-term feedback session^a^		
Yes	1307	78.8
No	352	21.2
Having a mid-term feedback session^a^		
Together with supervisor and nurse teacher	585	44.8
With supervisor	673	51.6
With healthcare educator	47	3.6
Achievement of clinical learning goals		
Very well	998	60.0
Fairly well	602	36.2
Moderately	57	3.4
Quite poorly	6	0.4
Very poorly	1	0.1
Supervision supported professional development^a^		
Very well	1131	68.1
Fairly well	429	25.8
Moderately	89	5.4
Quite poorly	11	0.7
Very poorly	1	0.1
Summative evaluation session at the end of the placement^a^		
Yes	1574	94.7
No	88	5.3
Summative evaluation session at the end of the placement^a^		
Together with supervisor and nurse teacher	673	42.9
With supervisor	868	55.3
With the nurse teacher	28	1.8
Theoretical studies supported learning in clinical practice^a^		
Very well	316	19.0
Fairly well	732	44.0
Moderately	487	29.3
Quite poorly	19	7.2
No theoretical studies before the placement	9	0.5
Recommendation of the clinical practicum placement to peer students^a^		
Very positively	1204	72.4
Positively	278	16.7
Probably yes	138	8.3
Probably no	35	2.1
No in any case	7	0.4

^a^Missing data

^b^Vocational training and qualifications for social and healthcare takes about 3 years to complete and does not fall within the EU directives

### Students’ perceptions of the student-patient relationship

The student-patient relationship was perceived as good. In more detailed examination, the mean scores over 8.1 were at very good level in six out of 13 items. Patients instructed students in care activities was the only item that was perceived to be at satisfactory level.

The internal consistency of the SPR Scale was evaluated calculating Cronbach’s coefficient alpha, which indicated acceptable reliability (Table 2).

**Table 2:** Students’ perceptions of student-patient relationship (*n* = 1664)

Brief description of item content of SPR Scale	Number of items	Cronbach’s α	Students (*n* = 1664) Mean^a^ (Sd)
**Facilitative student-patient relationship**	**13**	**.90**	**7.9 (1.6)**
Mutual understanding in students’ and patients’ best interest.			9.1 (1.5)
Student listened to patients’ concerns.			8.7 (2.0)
Student encouraged patients.			8.7 (1.9)
Student discussed confidential matters with patients.			8.5 (2.1)
Student talked with patients about their emotions.			8.4 (2.1)
Student’s actions were guided by patients’ wishes.			8.2 (2.2)
Student knew patients as individuals.			8.0 (2.5)
Patients as experts of their own situation.			8.0 (2.2)
Patients presented views about care.			8.0 (2.3)
Patients provided valuable information about illness.			7.8 (2.5)
Student acted as patient advocate.			7.7 (3.0)
Patients gave feedback on student actions.			6.5 (2.9)
Patients instructed students in care activities.			4.4 (3.2)

^a^Very weak = 0–2.0, Weak = 2.1–4.0, Satisfactory = 4.1–6.0, Good = 6.1–8.0, Very good (to be pursued) = 8.1–10

### Students’ perceptions of the quality of the CLE

In general, students were very satisfied with the quality of the CLE (Table 3). They perceived the pedagogical atmosphere, premises of care, premises of learning, and supervisory relationship as very good and the role of the teacher as good. The supervisory relationship was rated the highest. The five highest rated items were those in Supervisory relationship describing mutual respect and approval in the supervisory relationship (mean 9.2, SD 1.6), supervisors’ positive attitude towards supervision (mean 9.2, SD 1.6), students’ individual supervision (mean 9.2, SD 1.6) and mutual interaction in the supervisory relationship (mean 9.1, SD 1.7), and in premises of learning describing the use of patient cases in the supervisory process (mean 9.1, SD 1.6).

**Table 3:** Summary of students’ perceptions of the quality of CLE (*n* = 1664)

Dimensions of the quality of CLE	Number of items	Cronbach’s α	Students (*n* = 1664) Mean^a^ (Sd)
Pedagogical atmosphere	7	.88	8.5 (1.5)
Premises of care	4	.81	8.5 (1.4)
Premises of learning	7	.88	8.8 (1.4)
Supervisory relationship	8	.97	9.0 (1.6)
Role of the teacher	9	.93	6.9 (2.4)

^a^Very weak = 0–2.0, Weak = 2.1–4.0, Satisfactory = 4.1–6.0, Good =6.1–8.0, Very good (to be pursued) = 8.1–10

The role of the nurse teacher received the lowest ratings (Table 3). At item level, the five lowest rated items out of nine were those describing teacher’s ability to integrate theoretical knowledge into practice of patient care (mean 7.3, SD 2.5), teacher and clinical team working in supporting student learning (mean 7.0, SD 3.0), teacher helping the student to reduce the theory-practice gap (mean 6.8, SD 2.7), teacher’s expertise to the clinical team (mean 5.3, SD 3.2), and teacher as a member of the clinical team (mean 5.1, SD 3.2). Furthermore, the internal consistency of the CLES + T Scale indicated acceptable reliability (Table 3).

### The association between the student-patient relationship and the quality of the CLE

A statistically significant positive correlation was found between the student-patient relationship and the quality of the CLE. Spearman’s *r*-values ranged from 0.32 to 0.35 (*p* < .0001) indicating moderate positive linear correlation (*r* = 0.3–0.5) between the student-patient relationship and all five dimensions of CLES + T Scale (Table 4).

**Table 4:** Spearman’s correlations between the student-patient relationship and the quality of the CLE (*n* = 1664)

Dimensions of the quality of CLE	Student-patient relationship	
	r*	*p*-value
Pedagogical atmosphere	.35	*p* < .0001
Premises of care	.34	*p* < .0001
Premises of learning	.33	*p* < .0001
Supervisory relationship	.33	*p* < .0001
Role of the teacher	.32	*p* < .0001

*The Spearman’s correlation coefficient from .30 to .50 was interpreted as moderate positive correlation at the significance level of *p* < .0001

### Factors associated with the student-patient relationship and the quality of the CLE

The student-patient-relationship was perceived as significantly better among nursing, public health nursing, midwifery, paramedic nursing, practical nurse, physiotherapist, and occupational therapeutics students and those with more than 6 weeks’ clinical practicum compared to biomedical laboratory scientist students and students with 4–5 weeks’ clinical placement (*p*-values ranged from .05 to < .0001). Students who had a mid-term feedback session (*p* = .004) gave significantly higher scores on the student-patient relationship compared to those without this session. Students who achieved their learning goals during the placement very well perceived the student-patient relationship as significantly better compared to those who achieved their learning goals fairly well or moderately (*p*-values ranged from .006 to < .0001). Students perceived the student-patient relationship as significantly better if the theoretical studies supported their clinical learning very well or fairly well compared to students who reported moderate or quite poor support (*p*-values ranged from .025 to .008); the same was true for students with supervision supporting professional development very well compared to students with supervision supporting professional development quite poorly (*p*-values ranged from .037 to .008). (Table 5).

**Table 5:** Background factors related to the student-patient relationship and the quality CLE (*n* = 1664). Statistical procedure comprised multi-factor analysis of variance

Background factors	Student-patient relationship and the quality CLE	*P*-value*
Degree programme	Student-patient relationship	< .0001
	Premises of care	.023
Duration of clinical placement	Student-patient relationship	.030
	Pedagogical atmosphere	.022
	Premises of learning	.016
Occurrence of supervision	Pedagogical atmosphere	< .0001
	Premises of care	< .0001
	Premises of learning	< .0001
	Supervisory relationship	< .0001
Discussion about learning goals with a named supervisor	Pedagogical atmosphere	.031
	Premises of learning	< .0001
	Supervisory relationship	< .0001
Having a mid-term feedback session	Student-patient relationship	.004
	Role of the teacher	< .0001
Achievement of clinical learning goals	Student-patient relationship	< .0001
	Pedagogical atmosphere	< .0001
	Premises of care	< .0001
	Premises of learning	< .0001
	Supervisory relationship	< .0001
Supervision supported professional development	Student-patient relationship	.045
	Pedagogical atmosphere	< .0001
	Premises of care	< .0001
	Supervisory relationship	< .0001
	Role of the teacher	.001
Summative evaluation session at the end of the placement	Role of the teacher	< .0001
Theoretical studies supported learning in clinical practice	Student-patient relationship	< .0001
	Pedagogical atmosphere	.015
	Premises of care	< .0001
	Premises of learning	.005
	Role of the teacher	< .0001
Recommendation of the clinical placement to peer students	Pedagogical atmosphere	< .0001
	Premises of care	< .0001
	Premises of learning	< .0001
	Supervisory relationship	< .0001
	Role of the teacher	.020

*The significance level for *p*-values was set at .05

Several statistically significant factors associated with the quality of CLE were found (Table 5). As for degree programmes, occupational therapy students perceived the premises of care as significantly better than biomedical laboratory scientist students (*p* = .045). Pedagogical atmosphere and premises of learning were perceived as significantly better among students with a clinical practicum of 3 weeks or less than those with 4–5 weeks (*p*-values ranged from .027 to .017). Students with a named personal supervisor and a well-working supervision relationship regarded the pedagogical atmosphere, premises of care, premises of learning and supervisory relationship as significantly better than those with a named supervisor and poorly-working relationship (*p*-values ranged from .012 to < .0001). They also gave significantly higher ratings on the premises of learning than those with a group supervisor rather than an individual supervisor, as well as on the supervisory relationship than those with no named supervisor or whose supervisors varied according to shift or place of work (*p*-values ranged from .016 to .001) (Table 5).

Pedagogical atmosphere, premises of learning and supervisory relationship were perceived as significantly better among students who discussed their learning goals with a named supervisor compared to those who did not (*p*-values ranged from .031 to < .0001). Students who had a ‘half-way’ feedback session and students who had a summative evaluation session with their supervisor and teacher at the end of the placement perceived the role of the teacher as significantly better compared to those with no half-way feedback session and those with summative evaluation only with their supervisor, respectively (*p*-values (*p* < .0001). Students who achieved their learning goals during the placement very well regarded the pedagogical atmosphere as significantly more positive than those who achieved their learning goals fairly well or moderately whereas the opposite was true for premises of care, premises of learning and supervisory relationship (*p*-values ranged from .025 to < .0001). Students with supervision that supported their professional development very well regarded the pedagogical atmosphere, premises of care and supervisory relationship and the role of the teacher as significantly more positive than all the others (*p*-values ranged from .001 to < .0001) (Table 5).

Students with theoretical studies that supported their learning during the clinical practicum very well perceived the premises of care as significantly better than those with theoretical studies supporting their learning moderately (*p* < .0001). Furthermore, students with theoretical studies supporting their learning in clinical practice fairly well evaluated the pedagogical atmosphere, premises of care and premises of learning as significantly better than students with theoretical studies supporting their learning in clinical practice moderately (*p*-values ranged from .030 to .003). Students with any theoretical studies supporting their learning in clinical practice evaluated the role of the teacher as significantly better than those who had no theoretical studies before the placement (*p* < .0001) (Table 5).

The more positively the students recommended their clinical placement unit to their peers, the better they perceived the pedagogical atmosphere, premises of care, premises of learning, and supervisory relationship (*p*-values < .0001). Those students who recommended the clinical placement unit very positively to their peers also perceived the role of the teacher as significantly better than those who did not recommend the unit at all (*p* = .036) (Table 5).

## Discussion

This study examined the student-patient relationship, the quality of the CLE and associated factors as perceived by healthcare students. A positive and supportive pedagogical atmosphere in terms of interaction, behaviour, attitudes and values on the part of supervisors, other staff, teachers and patients characterise the quality of CLE and thus determine students’ learning experiences and clinical learning outcomes [11, 21, 24, 38]. In clinical education, emphasis on patient-centred care and enhancing patient autonomy and expertise in care are of great importance and thus shape the self-efficacy and competence of students in providing holistic healthcare [2, 47]. Therefore, the perspective of the student-patient relationship offers a more complete understanding of the context of CLE [4–6].

In this study, the student-patient relationship was perceived as good among students. The quality of CLE was also moderately associated with students’ perceptions of their relationships with patients, thus showing the student-patient relationship to be a core element of the CLE [6, 7, 24]. A sufficient and relevant theoretical basis of clinical practice, achievement of learning goals, and professional development supported by good quality supervision strengthen students’ self-confidence, comfort level and their satisfaction with relationships with patients [11, 13] and the quality of the CLE [20]. Special attention needs to be paid to students who face challenges in meeting their intended learning goals during their placement due to issues such as insufficient theoretical knowledge base. Besides clinical training, the insight and expertise of patients should be used actively in healthcare students’ theoretical studies in order to guarantee the best possible basis for patient-student interaction and patient-centred care in both single degree programmes and in interprofessional learning of healthcare students from more than one degree programme [11, 13].

Enabling and supporting the use of patients’ expertise of experience in a more active manner, especially in providing an illustration of their illness and care, thus boosting students’ understanding of what they have learned in theory, is of great importance [47]. By providing feedback on students’ performance, patients have an important role in shaping the competence priorities for future health professionals [5, 6, 33]. Experiences of patient relationships can also enhance students’ coping with the emotions and stress related to patient care while the role of supervisors and teachers is to help students deal with these emotions through debriefing [47, 48].

Longer practicum periods associated with better student-patient relationships allow better quality and more time being spent on direct patient contacts, and thus promote shared learning through dialogue between students and patients, benefiting both [5, 7, 12, 14]. In contrast to earlier studies, the length of the clinical practicum does not necessarily guarantee the quality of the CLE [18, 24, 40]. Therefore, clinical practicums planned collaboratively by students, supervisors and teachers that give students independence, responsibility and support their level of competence enhance meaningful learning experiences with patients [11, 15, 22]. Moreover, studies with long-term follow-up of the impact on student-patient relationships and the quality of CLEs are needed, especially in the clinical areas where patient-centred clinical education approach is used.

Overall, students’ perceptions of the CLE in terms of pedagogical atmosphere, premises of care, premises of learning, and supervisory relationship indicated good quality. Furthermore, the quality of the CLE was linked to students’ tendency to recommend their clinical practicum placement to peers [36] and may thus strengthen students’ professionalism and likelihood to stay in the health workforce [40]. This study revealed the complexity and multidimensionality of the CLE with a number of crucial elements affecting students’ perceptions of the quality of the CLE [49]. The results are in line with studies mostly conducted with nursing students [11, 17, 24, 40] but also among other healthcare students [15, 36]. The supervision relationship has especially been shown to be a central factor that determines the CLE both in hospital, home-based or primary health care [11, 22, 24, 38, 43, 50] and nursing home contexts [16, 22].

The findings that the quality of CLE is affected by a named supervisor and the supervision proceeding as planned [12, 15, 17, 18, 36, 40] confirms students’ preferences towards a personal supervisor. However, student-dedicated units with teamwork and collaboration between students under supervision, often from more than one professional discipline, combined with a patient-centred approach, have been shown to be a good alternative to individual supervision [7, 11, 12, 14]. Therefore, increasing patients’ engagement in shaping future health professionals’ competence to meet the health needs of patients also necessitates focusing learning on the student-patient relationships that are most desirable in interprofessional clinical learning environments with new coaching and supervising methods [2, 3, 47].

In line with other studies, the role of the teacher was perceived less positively than other dimensions of CLE [24, 36]. This may be because during clinical placements, face-to-face contacts with teachers have increasingly been replaced by the use of various online collaboration methods [20, 40]. Therefore, teachers are not necessarily regarded as members of the clinical care team, but regular contacts with them are considered important. Students need opportunities to reflect on their experiences with their supervisors and teachers, and they expect constructive feedback from both, not only at mid-term and final clinical evaluation, but also on an ongoing basis. Reflective discussions about individual learning goals and outcomes with the supervisor and teacher are essential for supporting students’ professional development and determine their experienced quality of CLE [15, 17, 36].

### Strengths and limitations

This cross-sectional study represented Finnish healthcare students in a variety of different clinical practicum placements in one university hospital district where patients have been actively involved as experts of experience in the design, planning and delivery of health services, including clinical training of healthcare students. A large study dataset (*n* = 1644) increased the study’s external validity; hence, the results may, with caution, be considered to be representative of the university hospital context in Finland. The questionnaire was not answered by all students having their clinical practicum. Thus, the students participating in the study might have caused a potential social-desirability bias, and the results might to some extent be specific to the sample.

Both scales were arranged on a 11-point Likert-scale so that the students could indicate their actual agreement scores. The scales included descriptive explanations of 0 = strongly disagree 10 = strongly agree, with labelled endpoints. The midpoint of the scale along with the endpoints would have decreased the number of nonresponses [51].

This study offers new insight into the healthcare student-patient relationship in the CLE context in the university hospital context. However, contextual differences in clinical practicum placements need to be considered. The university hospital district offers specialized medical care including outpatient clinics, inpatient wards and emergency units. Lack of unit-specific data regarding the students’ clinical placements and the quality and time spent on direct care contacts with patients of different ages, health status and admission reasons can be regarded as a limitation of this study and needs to be the focus of future student-patient relationship research.

Even though the sub-sample of laboratory scientist students was very small, the questions concerning the student-patient relationship were found inappropriate for students working with no or scarce contact with patients. Therefore, it would be wise to extend the use of the SPR Scale only to those clinical placements that involve direct patient care contacts or client service practices.

## Conclusions

Focusing on relationships with patients and the quality of CLE are crucially important for the professional development of healthcare students as they are expected to deliver high quality and patient-safe care after graduation. Therefore, the emphasis on student-patient relationships, as an untapped resource, deserves its full realisation in the CLE and the supervision of students, taking into account patients’ consent and respecting their voluntary participation. Collaboration between educational institutions and healthcare organisations is needed in establishing a safe and supportive CLE with the aim of promoting patients’ collaborative educational role and making it valued. Supervisors, for their part, need education and support on their role in the patient-centred approach in encouraging and supporting student learning, thus shifting the focus from themselves towards dialogue between students and patients.

## Data Availability

The data that support the findings of this study are available from the Helsinki and Uusimaa University Hospital District but restrictions apply to the availability of these data, which were used under license for the current study, and so are not publicly available. Data are however available from the authors upon reasonable request and with permission of the Helsinki and Uusimaa University Hospital District.

## Abbreviations

CLE: Clinical learning environment
SPR: Student-patient relationship
CLES+T: Clinical Learning Environment, Supervision and Nurse Teacher Scale
